# Toxical Effects of the Sulphate of Quinine

**Published:** 1845-06

**Authors:** 


*Toxical Effects of the Sulphate of Quinine.*—M. Desiderio found this substance, given in toxical doses to animals, to produce somnolence, a difficulty to stand, a tendency to remain in one position, defective vision, and lowering of the eyelids. Distilled water of the lauro-cerisus dissipates these symptoms; alcohol or acetate of morphia increases them. Bleeding is a very efficacious remedy.

